# How telemonitoring impacts the responsibilities of patients and healthcare professionals in cardiology: A Q-methodology study

**DOI:** 10.1177/20552076261484036

**Published:** 2026-08-28

**Authors:** Eva Meier, Marieke A. R. Bak, Job van Exel, Michiel M. Winter, Mariette van den Hoven, Tessel Rigter, Marlies P. Schijven

**Affiliations:** 1 Department of Ethics, Law & Humanities, Amsterdam UMC Location Vrije Universiteit Amsterdam, Amsterdam, the Netherlands; 2 Amsterdam Public Health, Digital Health, Amsterdam, the Netherlands; 3 Amsterdam Public Health, Personalized Medicine, Amsterdam, the Netherlands; 4 Department of Preclinical Medicine, TUM School of Medicine and Health, Institute of History and Ethics in Medicine, Germany; 5 Erasmus School of Health Policy & Management, 6984 Erasmus University Rotterdam, Rotterdam, the Netherlands; 6 Department of Cardiology, Amsterdam UMC Location University of Amsterdam, Amsterdam, the Netherlands; 7 Cardiology Centers Netherlands, Utrecht, the Netherlands; 8 Department of Community Genetics, Amsterdam UMC Location Vrije Universiteit Amsterdam, Amsterdam, the Netherlands; 9 Department of Surgery, Amsterdam UMC Location University of Amsterdam, Amsterdam, the Netherlands; 10 Department of Gastroenterology and Metabolism, Amsterdam UMC Location University of Amsterdam, Amsterdam, the Netherlands

**Keywords:** digital health, telemonitoring, cardiology, responsibility, ethics, Q-methodology

## Abstract

**Objective:**

Telemonitoring is widely used in cardiology but raises questions about the impact on roles and responsibilities for patients and caregivers. This study explores this using a Dutch cardiology telemonitoring program.

**Methods:**

Q-methodology was employed, combining quantitative and qualitative data from semi-structured interviews. Participants ranked statements on caregiver and patient roles and responsibilities in telemonitoring and explained their rankings. By-person factor analysis was used to identify clusters in the statement rankings. These factors were interpreted using interview data.

**Results:**

Thirty participants revealed four viewpoints on roles and responsibilities in telemonitoring: (1) *Balancing Act*, (2) *From Clinical Observers to Remote Monitors*, (3) *Isolated Responsibility*, and (4) *Responsible but not Alone*. All viewpoints agree on three aspects: patients are responsible for taking and submitting accurate measurements, that nurses’ roles are shifting, and that technology developers are accountable for device reliability while caregivers are responsible for data-usage in decision-making. Disagreements between viewpoints remain on four aspects: the extent of patients’ responsibility regarding understanding measurements and alarming caregivers, changes in nurses’ decision-making responsibilities, and how caregiving responsibilities are shared.

**Conclusion:**

Ambiguity about responsibilities create practical and ethical challenges, including increased workload for nurses, lack of overview among cardiologists, anxiety in patients, and inconsistent care delivery. Unrecognized or unaddressed differences in views about roles and responsibilities using telemonitoring can negatively affect patient care. Appropriate implementation and use of telemonitoring requires sound ethical prologue and dialogue, not only to sensitize stakeholders about differing interpretations of responsibility, but also to ensure a structured approach when implementing telemonitoring to support best care.

## 1. Introduction

Telemonitoring of patients at home is one of the most widely adopted digital health solutions, accelerated by the COVID-19 pandemic.^1,2^ It can be referred to as the delivering of care at home using wearables, sensors and mobile applications to monitor patients' health status, diagnose medical conditions and to adjust prescription regimen.^3^ Telemonitoring has become a mainstream technology in healthcare, especially in the domain of cardiology.^1,2,4^ An important reason is that numerous cardiac disorders, such as heart failure or arrythmias, can effectively be telemonitored.^4,5^ When appropriately used, telemonitoring devices can provide concise data that can be reviewed and managed remotely.^5^ Telemonitoring thus promises to reduce costs by shortening hospital stays, decreasing travel time for patients, and empowering patients to manage their own health.^6,7^ However, concerns have been raised about implicitly changing roles and responsibilities for both caregivers and patients when they use telemonitoring.^8^

Telemonitoring patients at home places both caregivers and patients in new roles, and each role comes with new responsibilities. Patients take their own measurements outside a clinical setting and share them digitally with their caregiver, either by manually or automatically uploading the data to their personal electronic health record (EHR). These tasks were previously exclusively performed by caregivers, often nurses. Consequently, nurses’ roles shift from being solely direct caregivers to also being technological gatekeepers. Their new role also focusses on instructing patients on setting up equipment, assessing data and deciding on follow-up actions. In addition, physicians are positioned in new roles too, focusing on validating data and deciding on appropriate follow-up actions remotely rather than during face-to-face consultations.^8^ It is important to recognize that new roles bring new responsibilities. When it is unclear which responsibilities belong to which role, the consequences can be severe. In practice, there must be a good understanding of who is responsible for accurate data entry, validation, and initiating appropriate follow-up care. For instance, the FDA revoked a home telemonitoring device after ECGs failed to reach treating cardiologists, resulting in two deaths and hundreds of health complications.^9^ This example highlights that such issues may very well occur because the shift in roles and responsibilities associated with telemonitoring is not debated or agreed sufficiently before it is implemented.^8^

Thus, the reshaping of roles and their associated responsibilities raises important ethical questions, such as: Should patients be held responsible for generating accurate data? Are caregivers accountable for decisions based on -potentially unreliable- home measurements? How can caregiving responsibilities be shared fairly and reliably in a remote setting? While these issues are mentioned in theoretical ethics literature and explored in a handful of empirical studies, findings are rather inconclusive.^10,11^ Hence, more in-depth research is needed on the shift in responsibilities when using telemonitoring in a real-world setting.

Our study aims to identify, categorize and understand the impact of telemonitoring in cardiology on the perceived roles and responsibilities of patients and caregivers from an ethical point of view. To this end, we examine a Dutch telemonitoring program launched in 2016 by a private outpatient clinic. This telemonitoring program has evolved into a widely adopted program serving around 4,000 cardiac patients in the Netherlands. Given its broad range of applications, including the monitoring of heart failure, arrhythmias and resistant hypertension, this seems a useful case study for examining the impact of telemonitoring on roles and responsibilities of patients and caregivers. Our primary research question is: *How do caregivers and patients perceive telemonitoring in cardiology affects their roles and responsibilities?* To explore this, we use Q-methodology, a mixed-methods approach that combines statement ranking with qualitative interview analysis.^12,13^ Our goal is to provide practical considerations for developing (implementation) guidelines, training of patients and caregivers, and mentoring programs to ensure the appropriate implementation and good use of telemonitoring in cardiology.

## 2. The telemonitoring program

**Figure 1.**
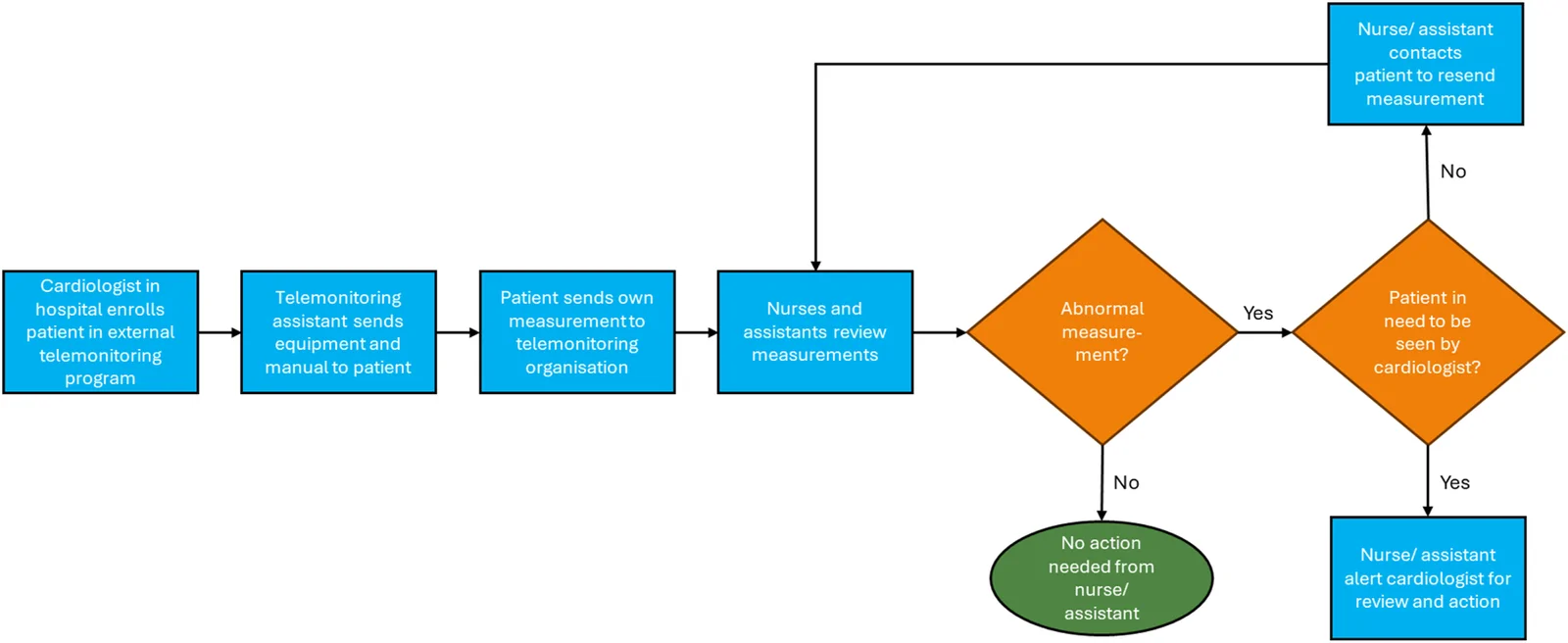
Simplified process of the telemonitoring program.

The telemonitoring program consists of three different care pathways: cardiac arrhythmia, resistant hypertension, and heart failure.^14^ Eligibility for each pathway is determined by the treating cardiologist in the hospital (Figure 1). The cardiologist can enroll patients in the telemonitoring program, which is a service provided by a private outpatient clinic operating independently of the hospital. The telemonitoring program operates on a 120-day subscription basis, costing €170 per period. It is covered by all Dutch health insurances with an annual deductible of at least €385. This deductible applies to all healthcare costs except general practitioner care. Therefore, patients must pay out-of-pocket until they exceed the deductible, after which insurance covers the costs.

**Figure 2.**
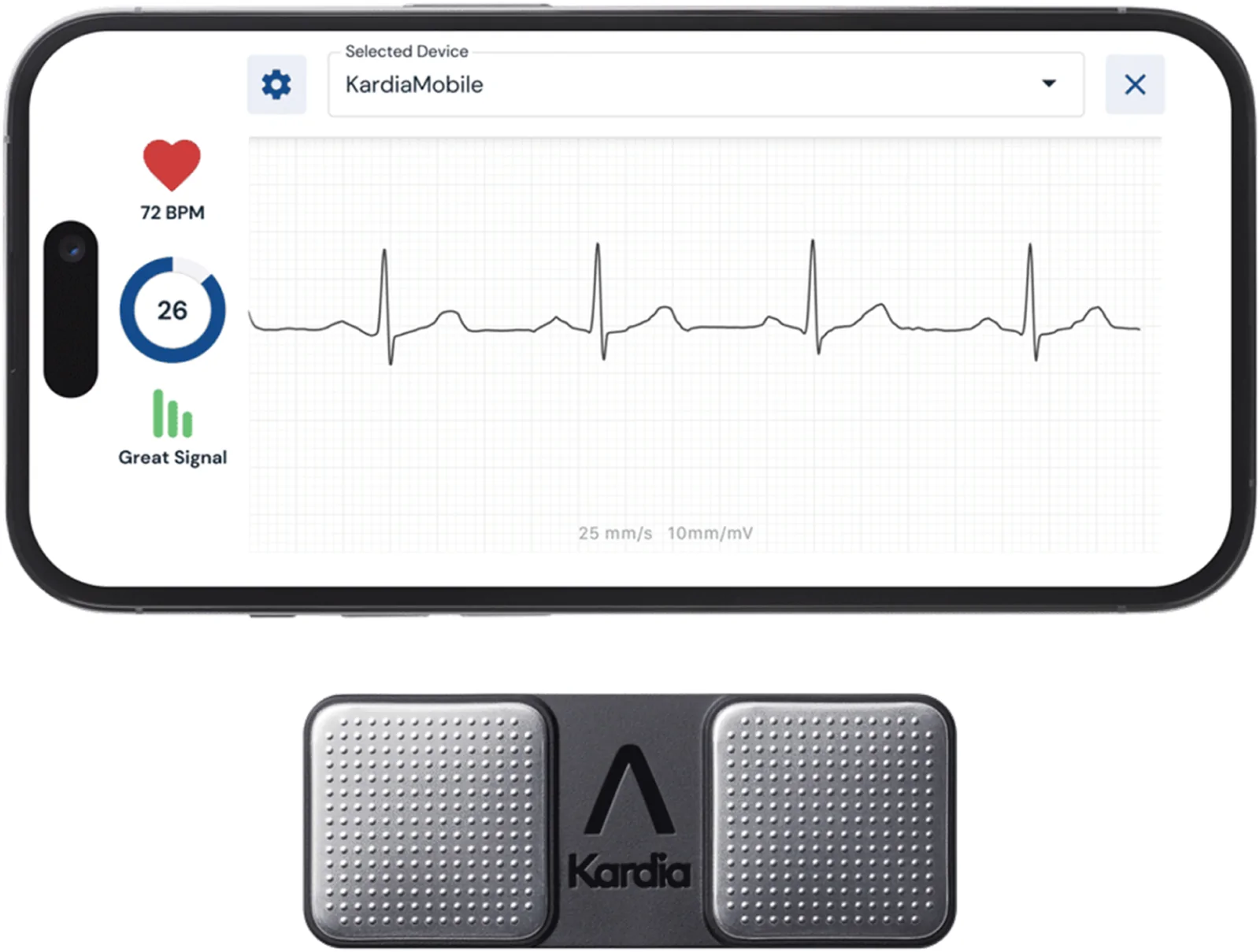
AliveCOR KaMobile (image source: https://kardia.com/products/kardiamobile).

The cardiac arrythmia program provides a diagnostic tool to follow-up for patients with atrial fibrillation (AF), palpitations of unknown origin or near-collapse.^15^ Patients participating in this program use KardiaMobile (KM, AliveCor, Inc. Mountain View, CA United States), an FDA-approved device to record a 30-second single-lead ECG by placing a finger on each of the two electrodes (Figure 2).^16^ In addition, patients must install AliveCor’s KardiaMobile app on a compatible personal phone or tablet. This app analyses the recorded ECGs using an algorithm that classifies them as normal sinus rhythm, potential AF, unclassified, or unreadable. Patients can then optionally describe how they felt at the time of the recording. So, patients do not have to enter the data manually, but the patient does have to actively tap ‘send’ to transmit the ECG to their EHR via the app. A dedicated telemonitoring team reviews all incoming ECGs using specific protocols. After evaluation, and depending on the results, a team member from the telemonitoring program contacts the patient within a working day and/or consults a physician if needed. ECG monitoring is available from Monday to Friday during regular office hours, so patients are advised to call 112 or go to the hospital in case of uncertainty or emergency.

The resistant hypertension program is designed to manage blood pressure by monitoring blood pressure at home, and providing targeted counselling. Patients who use at least three different classes of blood pressure medication, but remain hypertensive in the cardiologists’ office, are eligible for participation. In this program patients use the iHealth Track Blood Pressure Monitor, an FDA-approved device and must download the Heart for Health application on a compatible personal phone or tablet.^17^ Once connected, the monitor transmits the recorded blood pressure measurements to the application, that automatically forwards the data to their EHR. The assistants from the telemonitoring program review all incoming blood pressures using a set protocol. Depending on the evaluation of the measurements, actions may include waiting for further observation, conducting a telephone consultation with lifestyle counselling, or arranging a telephone consultation with the treating cardiologist at the hospital.

The heart failure program operates similarly to the resistant hypertension program but includes monitoring of patients’ weight using the iHealth Lina Scale. The weight measurements are also transmitted to the EHR and reviewed by the telemonitoring team.

Patients receive the medical equipment in accordance with the program to which they are assigned. Patients get the KardiaMobile, iHealth Track, and/or iHealth Lina by post on a loan base, along with online instructions.

## 3. Methods

**Figure 3.**
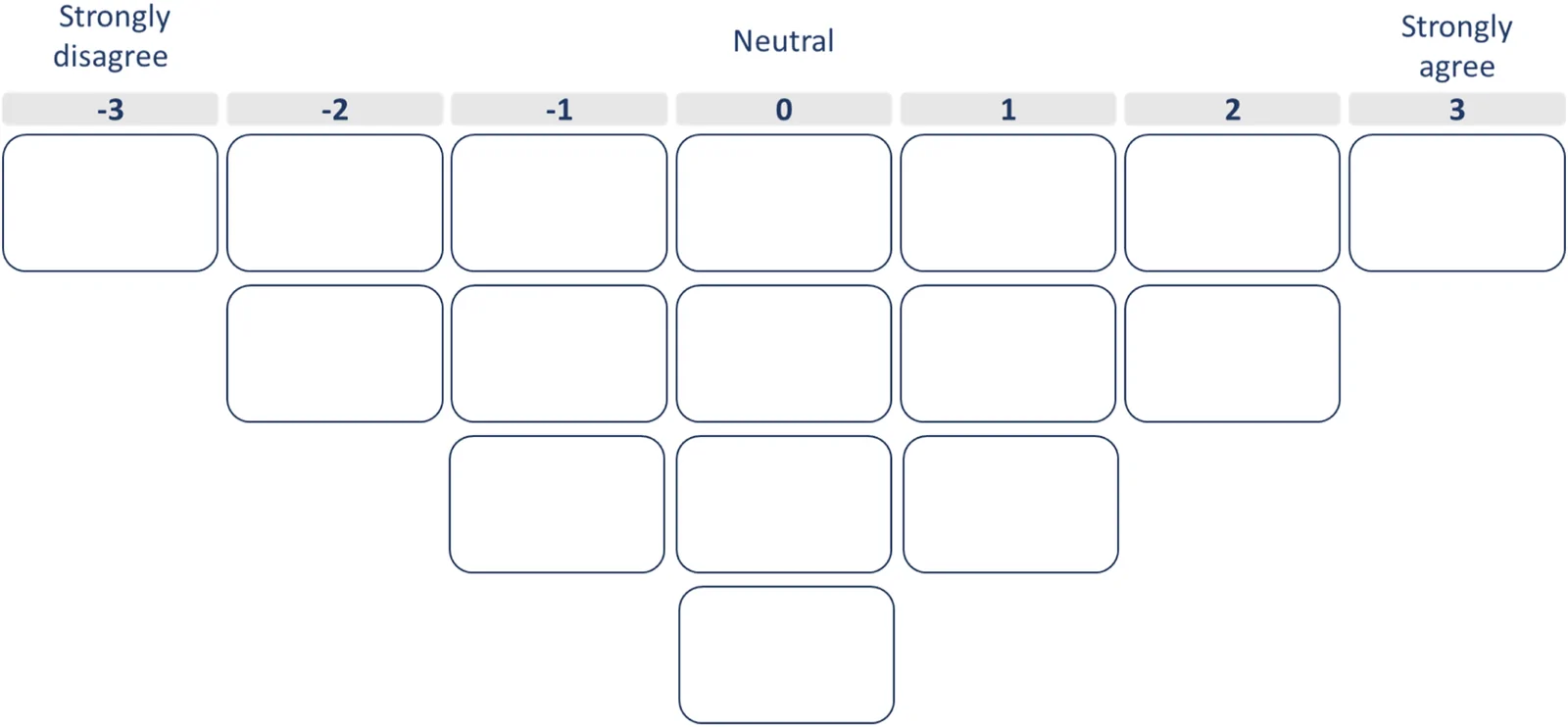
Forced sorting grid.

To investigate how telemonitoring in cardiology affects the roles and responsibilities of patients and caregivers, we used Q-methodology. From an ethical perspective, perceptions of one’s role and responsibilities are subjective and may vary among patients and caregivers. Q-methodology is well-suited to investigate perceptions in a systematic way, especially on complex and value-laden topics such as responsibility.^12^ This study was conducted in three steps. First, a comprehensive set of statements was generated representing the topic from all different perspectives. Second, participants were asked to rank the set of statements using a forced sorting grid from ‘strongly disagree’ to ‘strongly agree’ (Figure 3). During this step, participants were interviewed to explain their statement sorting. Third, the ranking of the statements was analyzed using by-person factor analysis to identify clusters of participants who had ranked the statements similarly. Finally, a narrative was developed for each factor, based on an idealized ranking of the statements and the qualitative data from the interviews. These steps follow the methodology checklist by Dieteren et al. (2023) and are described in more detail in Supplementary Table S1.^18^

### 3.1. Study design

We developed a set of statements to examine how telemonitoring in our case study impacts the roles and responsibilities of patients, caregivers and developers. To ensure relevance of the statements, we consulted a nurse and assistant from the telemonitoring team, reviewed caregiver protocols, and drew on results of a scoping review on digital health and moral responsibility.^8^ This process identified four themes: shifting responsibility to (1) patients, (2) nurses, (3) physicians, and (4) the establishment of collective responsibilities among all actors, including developers. The first author drafted 45 statements, which were subsequently refined through input from co-authors and a manager from the telemonitoring team. This process led to the removal of 20 negatively formulated statements (e.g. “the workload does not decrease for nurses”) that were flagged as redundant, as they conveyed the same meaning as their positive counterparts (e.g. “the workload increases for nurses”). After that, a pilot with a physician, nurse, and patient revealed that nine statements still lacked additional informational value. Given this and considering the time constraints of HCPs for the interviews, the final set was reduced to 16 statements in Dutch, which were randomly numbered for data collection and translated into English (Table 2). It is important to note that the ranking of the statements is only the first stage of the data collection; it is the basis for a follow-up interview, in which participants explain their ranking in their own words, giving meaning and adding nuance, enabling the researcher to understand their view. Ultimately, more statements means that participants spend more time ranking and have less time for the interview. Therefore, the set of 16 statements also created space for participants with busy schedules to elaborate on their experiences and add details they felt were would be missing from only inspecting the ranking of the statements. Based on our extensive qualitative material, we were therefore able to shed light on how participants interpreted these 16 statements.

### 3.2. Data collection and ethics statement

We used purposive sampling to capture diverse perspectives rather than represent population prevalence. Recruitment for this study took place between 6 March and 20 December 2024. Participants received study details beforehand and gave verbal or written formal consent. In cases of verbal consent, the consent statement was audio-recorded and transcribed. Data and names were pseudonymized, and the study was exempt from full ethics review (METC Amsterdam UMC, ref. 2024.0026). In individual interviews, participants read the statements aloud and ranked them on a forced-choice grid from ‘strongly disagree’ to ‘strongly agree’ (Figure 3), explaining their choices. They could review and adjust their rankings and were then asked to reflect on the statements they strongly agreed or disagreed with. The complete interview guide can be found in Supplementary S2. Interviews were recorded, transcribed, and returned for member checks.

### 3.3. Data analysis

By-person factor analysis was conducted in KADE v1.3.0 to identify clusters of participants with similar statement rankings and different form other participants. The first step was computing a correlation matrix between the individual rankings of the statements, indicating how similar these ranking were. This correlation matrix is subsequently subjected to factor analysis to identify a limited number of clusters of participants with similar statements rankings and different from other participants. We extracted the factors based on principal component analysis (PCA), followed by varimax rotation to maximize high and low valued factors.^19^ To determine the number of factors, we combined Goldberg’s (2006) method with qualitative interpretation.^20^ First, we looked at the correlations of the consecutive 1 to 6 factor extractions (S3 Fig. Factor flow diagram). When examining the correlations in the flowchart, it becomes clear that the factors from the 3-factor solution remain relatively consistent across the 4-, 5-, and 6-factor solutions, with correlations exceeding 0.80. Additionally, in the 4-factor solution, a new component (factor 4) emerges, with low correlations with the factors in the 3-factor solution. This component also remains stable in the 5- and 6-factor solutions, showing a fairly strong correlation of .73. In the 5- and 6-factor solutions, the new components were strongly correlated to other factors and added little distinctive meaning to the overall factor solution. Therefore, from inspecting the correlations between subsequent factor solutions, the 4-factor solution seemed the best starting point for interpretation, also involving the qualitative interview materials. Subsequently, we assessed the interpretability of each of the factors 4, in the 4-factor solution, which all appeared to have a coherent interpretation and to represent an interesting viewpoint on the topic. As a result, we selected the 4-factor solution, as the most meaningful reduction of the data, which accounted for a total explained variance of 59%. The analysis identified one respondent with a negative loading on factor 2. Given that this was the only negative loading on this factor, and the negative correlation between this participant and the other participants associated with this factor was only moderate (≤ -0.5), we excluded this participant from further analysis. Next, we computed factor arrays by calculating the factor scores for each statement as the weighted average ranking by the participants defining the factor. As a result, the factor array represents how a participant perfectly correlated with the factor would have ranked the statements. Finally, we interpreted the factors as viewpoints using characterizing and distinguishing statements for each factor and integrating qualitative interview data from participants defining the factor to check and refine the interpretation.

## 4. Results

A total of 30 individuals participated in our study, including patients, nurses, medical assistants, technicians, physicians and a manager of CCN (Table 1). We found four factors, of which the first factor explains the largest proportion of the variance in the data (21%), while the remaining three factors each account for 9%, 15% and 14% respectively. Of the 30 participants, 24 were significantly and uniquely associated with one of the four factors: Ten participants loaded on factor 1, two on factor 2, seven on factor 3 and five on factor 4 (S4 Table. Loading table). The interpretations of the factors, which we refer to as ‘viewpoints’, were based on their characterizing statements (i.e., the highest- and lowest-ranked statements; see Table 2), distinguishing statements (i.e., those ranked statistically significantly differently from the other factors; see Table 2) and the qualitative data of participants loading on the factors. Hereafter, we describe the four viewpoints in a narrative format. Each description is accompanied by two numbers: the first represents the statement number, while the second indicates its placement in the specific factor array (Table 2). The descriptions of the viewpoints are supported by illustrative interview quotes in Table 3.

**Table 1.** Demographic characteristics of participants.

Category	Total sample (N=30)
Age (years)	53.6
Sex %	
Female	57
Male	43
Role in telemonitoring	
Patient	9
Assistant	4
Nurse	5
Cardiologist	9
Technician	2
Manager	1
Years of experience (professionals)	13.1
Years of experience with telemonitoring (professionals)	4.1
Years of experience with telemonitoring (patients)	3.3
Digital literacy* (patients)	Adequate
Health literacy*^*^ (patients)	Adequate

*The digital literacy score is based on a 3-item measure scale, where the sum of the item scores indicates the digital proficiency of an individual, which is categorized into inadequate, marginal and adequate digital literacy.

**The health literacy score is based on the sum scores of the individual questions and subsequently categorized into inadequate, marginal and adequate health literacy.

**Table 2.** Overview statements and scores for the four viewpoints.

​	Statement	Viewpoint 1^	Viewpoint 2	Viewpoint 3	Viewpoint 4
1	Patients participating in the telemonitoring program can understand the measurements made.	-3*^*^	+2*	+3*	-1*^*^
2	The patient is responsible to take proper measurements.	+3	0*	+1*	+3
3	The involvement of the patient’s relatives, friends, or neighbors plays a crucial role in ensuring the successful operation of measurements.	+2	0	-1	-2
4	The use of telemonitoring makes the patient responsible for managing their own disease.	+1	0	0	+1
5	The patient has the responsibility to alert a caregiver when a measurement deviates.	-2	-2	-3	+2*^*^
6	The patient must perform a measurement at a fixed time. This increases his/her level of responsibility.	0	+1	0	0
7	The use of telemonitoring reduces the workload for the nurse.	-1*^*^	+1	0	+1
8	When using telemonitoring the nurse takes on more responsibility for medical decisions, and the physician has less.	0	+1*	-2*^*^	0
9	In case of abnormal measurements, the nurse decides whether additional examination is necessary.	0	+2	-1*^*^	+2
10	The use of telemonitoring changes the role of the nurse in patient care.	+2	+3	+1	0*^*^
11	It is the responsibility of the physician to assess whether a patient can participate in telemonitoring.	+1	-1*^*^	+2	+1
12	The implementation of telemonitoring reduces the workload of the physician.	+1	-1*	+2	0*
13	The use of telemonitoring reduces the physician´s involvement with the patient.	-1	-1	-1	-3*
14	When the physician can continuously monitor the patient’s measurements, he/she has more responsibility for the patient care.	-1	0	0	-1
15	When an error in the measuring equipment leads to an incorrect medical decision, the responsibility lies solely with the developer of the device and not the caregiver.	0	-3	+1	-2
16	If an error in the measuring equipment leads to an incorrect medical decision, caregivers are equally responsible as the developer.	-2	-2	-2	-1

^Viewpoint 1: ‘Balancing Act’; Viewpoint 2: ‘Nurses: From Clinical Observers to Remote Monitors’; Viewpoint 3: ‘Isolated Responsibility’, and Viewpoint 4: ‘Patients: Responsible but not Alone’.

*Distinguishing statements with significance at p<0.05 for the specific factor.

**Distinguishing statements with significance at p<0.01.

**Table 3.** Illustrative interview quotes supporting the four viewpoints.

Description of changing roles and responsibilities	Illustrative quotes
**Viewpoint 1**	
Patients’ responsibility for taking accurate measurements	*The patient is responsible because if you don’t get accurate measurements, you can’t do anything with them. So that’s the patient’s responsibility.* (R3, Nurse)
	*You don’t need to understand the measurements in detail, but you do need to understand a little bit about what you’re doing.* (R27, Cardiologist)
Shared responsibility with friends or family	*Sometimes, people don’t have children, a partner, or anyone around them except maybe a housekeeper. In those cases, they can’t manage the system and often have to be unenrolled. (*R9, Assistant)
Responsibility shift towards nurses for medical follow up	*Some patients find reassurance in the system, but others become so fixated on their heart that it turns into an obsession. In such cases,* [telemonitoring program] *does not help—they become completely agitated. So, it’s not always a positive effect. And you don’t know this beforehand. For example, the cardiologist gives a Kardia* (see Figure 2), *but then some patients call saying, “I’m getting anxious because of this.”* (R3, Nurse)
Role responsibility of physicians to explain the telemonitoring program	About statement 11: *Yes, and sometimes that’s done a bit carelessly (…) due to haste. The cardiologist might think, “Oh well, anxious patients, just quickly give them a Kardia* (see Figure 2)*.” That might sound a bit negative, but at least then they’re out of the consultation room.* (R3, Nurse)
	*(…) Sometimes you just don’t really know what exactly is needed to be able to do telemonitoring. And of course,* [telemonitoring program] *itself does know that. Because we’ve never actually seen or set it up physically ourselves. So it’s like: we just press a button. Sure, a blood pressure monitor is very straightforward, and I have seen a picture of that before, but I’ve never— and I know they need a specific kind of smartphone, but which one exactly, that also changes over time. So yeah, I always think: well, the telemonitoring program can figure that out themselves then*. (R23, Cardiologist)
	*It shouldn’t be that she* [referring to cardiologist] *feels like I’m going behind her back, arranging all sorts of things. If I were to just call* [telemonitoring program] *directly, like: “Hey, did you receive my measurements?”- I don’t really think that would be proper. As if I’m acting behind her back.* (R21, Patient)
Increased workload of nurses due to uninformed patients	*Patients are not always well-informed. Some assume that it’s offered as a free service and believe there are no costs involved. Cardiologists often don’t know the details either and sometimes tell patients that there are no costs. As a result, patients are misinformed from the very beginning, which I find frustrating.* (R9, Assistant)
Changing role of assistants and nurses due to commercial interests	*Before this, I always worked in elderly care, with people with dementia. (…) Back then, I was really in hands-on care, with lots of irregular hours, and that’s why I came here* [to the telemonitoring program]. *But to be honest, this is a commercial company, and that sometimes bothers me. In my previous job, I really felt at the end of the day that I had made a meaningful contribution, and now I feel that less.* (R9, Assistant)
**Viewpoint 2**	
Changing role of nurses	About statement 8: *I think it requires a different kind of skill set. You also see people less, you have less information. But then again, you also get feedback on how they’re doing more quickly, right? So that makes it easier in a way. I think you have to steer based on different indicators, so to speak. You have to navigate using different coordinates or beacons. So, some things are easier because you might get feedback again a week later. And other things will be a bit more difficult because you have less information*. (R20, Cardiologist)
Shared responsibility between patients and caregivers for measurements	*The patient has responsibility within their own space, and that should be encouraged. But if a measurement deviates, feedback should come from the caregiver, not the patient. Otherwise, you risk differences between assertive and shy individuals, creating inequities.* (R20, Cardiologist)
	*It’s also about self-management and taking responsibility for your own body and your own health. Well, the patient is competent enough to perform good measurements, and that competence should then be assessed during the introduction* [of the telemonitoring program]. (R15, Patient)
Physicians are less responsible than developers for the technology	*You also assign responsibility to the tech industry to ensure proper use, including implementation in hospitals, data processing, and user training. (…) And if most of the responsibility is placed on the developer, it shifts from being about the device itself to purchasing a service. But I still believe this does not absolve healthcare providers from their duty to take responsibility as well.* (R20, Cardiologist)
**Viewpoint 3**	
Patients’ responsibility for taking accurate measurements	*I always say: ‘measure three times to be sure,’ right? (…). Sometimes the battery of the device might be dead, that has happened to me too. Then it gives strange readings(…). I had a housemate, and I would take a measurement with them and think: is the device working properly? I’ve had those doubts when the reading was that far off, you know. Then it shows you an error code. And then you can look up what that error code means. Then you just look it up in the manual. Exactly, so then you just need to replace the batteries*. (R28, Patient)
	*If you make them responsible for administering chemotherapy, for example, that might be a bit too much. But measuring blood pressure is a responsibility most people can handle, so it works well and doesn’t compromise the quality of the measurements*. (R13, Cardiologist)
Patient responsibility will improve compliance	*People who see their own blood pressure in the telemonitoring system will have better compliance than when measurements are done anonymously every now and then at a general practitioner’s office or by a specialist. If you give people more insight into their own data, it improves compliance.* (R24, Cardiologist)
Responsibility of nurses to be transparent about the protocol	*You have slightly abnormal values, seriously abnormal ones, and minor deviations. Patients need to know that they won’t be called immediately for minor deviations. But for a significant abnormality, you need to tell them they will be contacted. So, the protocols for this must also be communicated to the patient. It must be clear to the patient what to expect, including whether or not a response is anticipated for a particular measurement.* (R24, Cardiologist)
Shift of nurses’ roles towards protocol duties	*Well, in principle, I do signal things, but I don’t make medical decisions. I signal them and pass them on to a doctor, like: ‘I see this or I see that.’ But (…) I don’t make the medical decisions. For example, if I see that they have certain arrhythmias, then the doctor wants to know that, so I forward it to the doctor. And I do talk with the patient, but I’m not the one who prescribes medication or anything like that.* (R7, Nurse)
	*It’s purely protocol-based. If something deviates from the protocol, they alert the doctor. (…) So, the responsibility does not lie with the nurse.* (R13, Cardiologist)
Navigating between protocol and perceived responsibilities	*So I forward it to the cardiologist who knows the patient: ‘Please review, because I see this and that, and adjust the treatment if necessary. Then I got a message back from a nurse in Zeeland saying: “We don’t have a cardiologist today, is it urgent?” Well, then I think: now I’m being given the responsibility of deciding whether it’s urgent or not.* (R7, Nurse)
Individualized patient responsibility	*If you say ‘turn right’ and I see a ditch there, then I’m not going to turn right. No. I believe you always have to keep thinking for yourself and stay realistic. I think if something doesn’t feel right to me, then I report it to* [the telemonitoring program] *. And then, yes, they do something about it. So, I think it’s also partly a matter of personal responsibility.* (R28, Patient)
Cardiologists are not accountable for reliability of technology	*We once used a Holter interpretation system, but it failed to detect a very serious condition. We immediately discontinued it. While I did feel responsible, ultimately, the developer bears the accountability.* (R13, Cardiologist)
**Viewpoint 4**	
Patients’ responsibility to act upon deviating measurements	*I really appreciate when people leave a comment because then you understand why the patient is sending in this recording. Sometimes you see ten recordings in a row, a rhythm strip with a perfectly normal rhythm, and then you think: why are they doing this? But if they include something like, ‘I don’t feel well because I’m dizzy,’ then you can follow up and think: maybe I should ask what’s going on or if something else might be wrong. But if you receive ten strips with nothing abnormal and everything looks fine, I wouldn’t call that patient*. (R10, Nurse)
	*You’re still responsible for your own heart rate, right? If my heart is acting up and I see it on my own device, and when it’s over, I press the cross and it gets sent off* [to the telemonitoring program]*. Then I’m still responsible myself, aren’t I?* (R17, Patient)
Change of nurses’ role but no responsibility shifts	*It’s a different role, but on the other hand, you still remain the eyes and ears of the doctor. So, yes, the role is slightly different because it’s remote (…). So it’s not the same as standing at the bedside; it’s actually monitoring. So yes, it’s a different role, but something that’s in line with the skills of a nurse.* (R11, Cardiologist)
	*There is now a much greater chance of capturing data because patients can record measurements at home.(…). So, it’s just a different way of providing care.* (R10, Nurse)
No impact on the patient-physician relationship	*The doctor sees the patient less often, but he is still involved. The patient will continue with regular check-ups. Yes, the doctor is less engaged, but he also has fewer unnecessary tasks to perform.* (R10, Nurse)
No shared responsibility with family/friends	*Look, if I couldn’t do it myself, (…) my wife could. Then I’d place my fingers on it, get the recording, and then she would have to see how my heart is doing. And I think, (…) they don’t need to know that. (…)I think: every patient should take care of knowing this for themselves.* (R17, Patient)
Collective responsibility for medical decision making with telemonitoring	*There can be an accumulation of factors. The patient might have carried out the instructions incorrectly, the nurse might have assessed it differently, and then decided whether or not to refer it to the cardiologist—perhaps due to an incorrect measurement or a misinterpretation. So, I don’t think there is a single person who holds sole responsibility for that.* (R10, Nurse)
	*There is always someone involved, and yes, if something goes wrong, it can also be a process. That’s often how it is with mistakes in healthcare; it’s not just one thing, but usually a series of events.* (R11, Cardiologist)

### 4.1. Viewpoint 1: Balancing act

The central focus of this viewpoint, on how caregivers and patients perceive telemonitoring in cardiology to affect their roles and responsibilities, is the moderate responsibility shift between patients and caregivers. According to this viewpoint, the delivery of care with telemonitoring is considered a shared responsibility. There is agreement that using telemonitoring shifts responsibility to patients for managing their own disease (4; +1; i.e., statement number 4, grid placement +1). Patients have the responsibility for taking accurate measurements, as this is essential for telemonitoring to function effectively (2; +3). However, this viewpoint distinguishably recognizes it as a shared responsibility with the patients’ network (3; +2), since many patients may not understand the measurements made (1; −3**). Participants associated with this viewpoint report that many patients, particularly older ones, rely on family members or friends for support when they struggle to set up the equipment or take measurements themselves.

Since patients may not understand the measurements made, they can also not be expected to act on deviating measurements (5; -2). This shifts some of the responsibility for providing the necessary follow-up to assistants and nurses. It highlights the impact of the changing role of nurses on being the first point of contact for patients and determining how to respond to their measurements (10; +2).

Distinguishing for this viewpoint is the perceived shift in workload from cardiologists to nurses (7; -1** and 12; +1). Guiding patients in the telemonitoring program and providing follow-up to measurements increases the burden on nurses, while cardiologist can shorten their consultations with patients. In this context, the placement of statement 11 in this viewpoint is interesting (11; +1). Some participants loading on this viewpoint –two assistants, two cardiologists and a patient– agreed with statement 11, suggesting that they view patient enrolment as the cardiologist’s responsibility, because they are the main points of contact for patients and can therefore make the most informed decision. However, others –two cardiologists– disagreed with this statement, as they claimed to have a limited understanding of the telemonitoring system, insisting that responsibility for its operation should lie with the program itself. This reflects conflicting expectations between those involved about who is responsible for enrolling patients in the telemonitoring program and informing them about the process of it and especially its costs.

This viewpoint captures a moderate responsibility shift towards patients, an increasing burden on nurses, and conflicting expectations among caregivers regarding enrolment responsibilities in telemonitoring. Therefore, it was labelled “Balancing act”.

### 4.2. Viewpoint 2: Nurses - from clinical observers to remote monitors

Participants associated with this viewpoint see three major changes in the roles and responsibilities of nurses (10; +3, 9; +2, and 11; -1**). First, as care tasks are now delivered remotely, nurses are described to have limited opportunities for direct clinical observation (10; +3). As a results nurses are seen in a new role, which requires them to take on greater responsibility for medical decision-making (8; +1*). However, nurses do not perceive this increased responsibility as adding to their workload (7; +1). Second, in this viewpoint there is agreement that responsibility for decision-making shifts towards nurses when abnormal measurements occur in telemonitoring, as they are the first to identify these changes (9; +2). Third, in contrast to viewpoint 1, participants holding this view agree on the role of nurses in determining patient eligibility for the telemonitoring program (S5. Factor arrays, Figure 2(a)). Since nurses often spend more time with patients, they have more insights into patients’ motivation and digital literacy. Therefore, decisions about patient inclusion into the telemonitoring program should be made collaboratively by nurses and cardiologists, rather than being solely the cardiologist’s responsibility (11; -1**).

In addition to the changes in nurses’ responsibilities, this viewpoint recognizes that multiple responsibilities are shared within the telemonitoring program. First, patients are not solely responsible for taking accurate measurements (2; 0*) or notifying healthcare providers about deviations (5; -2). Instead, these responsibilities are shared with caregivers in order to prevent inequitable care delivery between assertive and shy patients. Second, while participants associated with this viewpoint agree that patients are responsible for understanding the measurements (1; +2*), nurses are responsible for guiding and monitoring them, and cardiologists are responsible for re-evaluating the patients’ measurements. The cardiologist’s responsibility to check patients’ measurements, is described to lead to greater involvement of the cardiologist in patient care through telemonitoring (13; -1). Finally, incorrect medical decisions caused by faulty equipment are not the sole responsibility of developers (15; -3), or cardiologists (16; -2), but are shared by both actors.

This viewpoint is distinguished by the agreement on the major responsibility shift towards nurses. Therefore, it was labelled “Nurses - from clinical observers to remote monitors.”

### 4.3. Viewpoint 3: Isolated responsibility

At heart of this viewpoint is the recognition that using telemonitoring involves assigning individualized roles, each with a distinct set of responsibilities. Participants of this viewpoint agree that most patients understand the measurements (1; +3*), and are therefore expected to take them correctly (2; +1*). They see patients taking individual responsibility for their own measurements as a positive development because they believe that seeing their own measurements enhances compliance with their treatment. This contrasts with viewpoint 1, where patients are generally described as not being able to understand the measurements (S5. Factor Arrays, Figure 1(b)). However, this shift of patients’ responsibilities has clear limits. Similar to viewpoint 1, patients are not considered responsible for alerting caregivers about abnormal measurements (5; -3). Instead, only caregivers are thought to be responsible for the follow-up on patients’ measurements.

Distinguishing for this viewpoint is the agreement that nurses’ responsibilities are delineated within established protocols and that they do not decide on follow-up examinations (9; -1**). Consequently, their role is described to not shift toward greater medical decision-making (8; -2**) but becomes more administratively protocol-based. However, those holding this viewpoint also see that the changing role of nurses (10; +1), can sometimes create tension between protocol-based responsibilities and perceived responsibilities.

In terms of the impact on the roles and responsibilities of cardiologists, telemonitoring is seen as an enhancement of their medical decision-making responsibilities, as it provides more insights into patient data and increases their engagement in patient care (13; -1). Additionally, there is agreement that cardiologists must be able to trust the technology, and if a medical error occurs due to a technical failure, responsibility should lie with the developer and not the cardiologist (15; +1, 16; -2).

This viewpoint describes the roles of patients and caregivers individually, emphasizing their distinct responsibilities. Therefore, we labelled this viewpoint “Isolated responsibility”.

### 4.4. Viewpoint 4: Patients - responsible but not alone

Participants associated with this viewpoint see that telemonitoring in cardiology affects mainly patients’ role and responsibilities. Distinguishing for this viewpoint is the agreement that patients are expected not only to take accurate measurements (2; +3) but also to alert caregivers when results deviate (5; +2**). Patients are described to be responsible to provide contextual information, which nurses consider crucial for deciding on follow-up (9; +2). In contrast to viewpoint 1, those holding this viewpoint agree on the importance of self-reliance of patients (3; –2) (S5. Factor Arrays, Figure 1(c)). A patient associated with this viewpoint prefers to take responsibility for himself, managing the telemonitoring tasks independently and without support from his network (see Table 3).

In contrast to the major shift in patients’ responsibilities, participants holding this viewpoint feel that telemonitoring only subtly affects the roles of nurses and cardiologists (10; 0**, 8; 0, and 13; -3*). Nurses continue to provide patient care, but now do so remotely rather than face-to-face, resulting in less direct interaction. This is not seen as a fundamental change in their role (10; 0**, 8; 0). Similarly, cardiologists have less direct contact with patients, yet this reduction is not viewed as deteriorating the physician–patient relationship (13; -3**).

Finally, this viewpoint recognizes that medical decision-making is shared among the caregivers that are connected via telemonitoring (15; -2). Therefore, no single healthcare professional is solely responsible for an incorrect medical decision when there is an error in the measuring equipment.

This viewpoint thus mainly emphasizes the changes in roles and responsibilities of patients, while describing collective responsibility of caregivers. Therefore, we labelled this viewpoint: “Patients - responsible but not alone.”

## 5. Discussion

This study identified four distinct viewpoints among patients and caregivers in cardiology on how they perceive their roles and responsibilities when using telemonitoring (Table 3). All four viewpoints recognize that, in practice, patients are responsible for taking and submitting accurate measurements, that the role of nurses is reshaped, and that device developers bear responsibility for ensuring the reliability of the devices used in telemonitoring. However, the viewpoints diverge on multiple aspects. Notably, the scope of patient responsibility for providing timely and accurate measurements is contested, as views differ on whether patients understand the measurements made and whether they should actively notify HCPs in case of deviating measurements. There is also disagreement about the extent of nurses’ responsibility in medical decision-making and how responsibilities are shared between caregivers, patients and developers. Hereafter, we discuss whether and why these differences between viewpoints may cause problems for appropriate implementation and usage of telemonitoring, and how these could be addressed.

### 5.1. Consequences of differing viewpoints

#### 5.1.1. Challenge 1: Ambiguity in the scope of patients’ and nurses’ roles and responsibilities

This study demonstrates that telemonitoring does not lead to a uniform shift of nurses’ nor patients’ roles and responsibilities. Several viewpoints emphasize a shift in nurses’ roles and responsibilities that aligns with existing literature on telemonitoring. Using telemonitoring transforms nurses’ roles by replacing face-to-face nurse–patient interactions with communication via phone or email, increasing reliance on patient-generated data and increased responsibility in decision making.^21–24^ However, one viewpoint in our study does not perceive such a change in role or responsibility. This finding nuances the narratives of the impact of telemonitoring by showing that the shift in role and responsibilities is not just a uniform shift inherent to the technology itself, but is interpreted and enacted differently in practice, even within the same telemonitoring program and organization.

Importantly, this ambiguity in nurses’ roles and responsibilities extends beyond the nurses practice and directly shapes how patient responsibility is constructed. Much of the telemonitoring literature presents telemonitoring as enhancing patients’ autonomy, self-management, and responsibility for their own health, with patients positioned as capable interpreters of their measurements and active participants in their care process.^25–27^ Our findings partly align with this narrative. One viewpoint (viewpoint 4) reflects the figure of the “responsible patient”, who actively interprets data, makes independent decisions, and is willing to question or deviate from professional advice when it does not align with their lived experience. In this sense, telemonitoring can indeed function as an empowering technology for some patients. However, the other viewpoints challenge the assumption that telemonitoring uniformly empowers patients. Instead, they show that telemonitoring may place substantial and sometimes undue responsibility on patients who are unable to fully understand the measurements or their implications. Despite this limited understanding, patients sometimes are implicitly expected to act upon the data, to recognize when values are concerning, or to provide contextual explanations. As a result, responsibility is shifted onto patients without corresponding support, understanding, or agency. In these cases, telemonitoring places undue responsibility on patients rather than fostering empowerment.

Crucially, our findings suggest that the extent of patient responsibility depends on how nurses perceive their roles and responsibilities. Depending on their viewpoint, nurses may treat patients as active and responsible actors, expecting them to ‘signal’ how they feel alongside standardized measurements. Alternatively, they may see patients’ dependence on (informal) caregivers and recognize the need to fulfil a more supportive role. Patient responsibility thus emerges as relational and situational rather than inherent to telemonitoring.

By linking nurses’ role ambiguity to the construction of patient responsibility, our study adds nuance to existing debates about the impact of telemonitoring on nurses’ and patients’ roles and responsibilities. Rather than a straightforward shift of responsibility from nurses to patients, our findings suggest an uneven process in which responsibilities are implicitly enacted in everyday care practices. This relational dynamic may result in inconsistent expectations and unequal care delivery. Recognizing and addressing these interdependencies between nurses role interpretations and patient responsibility is therefore crucial for ensuring consistent and fair care delivery with telemonitoring.

We suggest that defining what types of data are considered valid for nurses (i.e., what constitutes “good enough” information), and also how these should be submitted by patients, as an important step to acknowledge the complexity of the varying experiences of the roles and responsibilities shift. Insufficient guidance may otherwise result in placing undue responsibility on patients and nurses.^28,29^ A promising framework for this purpose is the concept of Entrustable Professional Activities (EPAs), which were originally introduced to define clinical competencies.^30^ EPAs, which have already been proposed to guide the responsible use of AI in medicine, could be adapted for telemonitoring, outlining the specific responsibilities and competencies required of caregivers and patients alike to ensure its appropriately use for good patient care.^31^ This process should be integrated into caregiver training to support continuous reflection and improvement in telemonitoring programs.

#### 5.1.2. Challenge 2: Cultivating sensitivity to diverging views on collective responsibility

Although existing literature on telemonitoring recognizes the importance of collective responsibility, it provides little empirical insight into how collective responsibility is understood and enacted in practice.^32,33^ In this study we demonstrate how differently various actors perceive collective responsibility within telemonitoring, thereby shedding light on its practical and ethical implications. Collective responsibility arises when actions result from group interaction or collaboration, which is typically the case in healthcare.^34,35^ In our earlier review on responsibility and digital health technologies, we highlighted the need to focus on collective responsibility because “the framing of responsibility as something applicable to individuals only (…) separates health from its broader social, political and cultural components” (p. 28).^8^ While ethical discussions on responsibility commonly involve theoretical questions about directionality, the conditions for responsibility, and the role of free will, these issues lie outside the scope of this study.^34^ Instead, we examine how patients and caregivers, who form a collective using telemonitoring, perceive collective responsibility. In the case of the telemonitoring program, collective responsibility emerges as physicians, nurses, assistants, developers, managers and patients along with their social networks, collaborate to deliver patient care via telemonitoring.

The four viewpoints revealed different conceptions of collective responsibility, which leads to challenges at two moments. First, a lack of clarity about who is responsible for determining eligibility for the telemonitoring program and explaining it to patients, increases the workload for nurses and assistants. They must handle patients who are often unaware of potential out-of-pocket expenses and sometimes anxious about the care process. Second, lack of clarity on who in the collective is responsible for follow-ups, leaves cardiologists uncertain about the next steps to take, which potentially undermines consistent and reliable care. These issues ultimately reflect ethical tensions around how collective responsibility should be organized.

Differing interpretations of collective responsibility highlight a long-standing issue in bioethics: how can collective responsibility be defined?^35–38^ The main debate is whether collective responsibility is simply the sum of each individual´s responsibility within the collective, whereby each actor is responsible for their own actions, or whether the collective can be seen as an independent actor with its own identity and objectives, capable of bearing responsibility as a whole^.^.^35–38^ These conflicting interpretations are also reflected in the viewpoints, each offering a different understanding of ‘the collective’, its goals, and the balance between individual and collective responsibilities. Viewpoint 4 describes joint responsibility solely among healthcare professionals, forming a collective to provide care, excluding patients from the collective. Viewpoint 1 adds nuance to this interpretation of ‘the’ collective, by further distinguishing between the collective responsibility of caregivers, whose goal is to provide care, and the telemonitoring program itself, which is described as a commercial entity with an additional, business-oriented objective. Here, the telemonitoring program is not viewed as part of the caregivers collective. In contrast, Viewpoint 3 offers a more individualistic interpretation, where collective responsibility is understood as the sum of the individual responsibilities of patients and caregivers, each responsible for their own independent actions. This divergence, however, does not imply that one view is more valid than the other. In our view, collective responsibility goes beyond the sum of individual responsibilities. We believe that ‘the collective’ itself has a responsibility that relates to a group’s culture, which shapes the actions of individuals within the collective. Thus, rather than forcing alignment on a single view of collective responsibility, it is important to recognize and highlight these different aspects.

The telemonitoring set-up examined in this study was offered through a medical specialist, the cardiologist, which represents just one of many possible organizational models for this technology. In fact, telemonitoring can be organized in widely varying ways, and the roles and responsibilities experienced with the use of it shift accordingly. For instance, in the Netherlands, telemonitoring is often used in prevention or monitoring chronic conditions, which is care coordinated by general practitioners (GPs) or primary care centers.^39^ In those contexts, GPs, care center staff or family members might take on roles and responsibilities comparable to those described here by the nurses and patients. Consequently, this underscores that the roles and responsibilities experienced when using telemonitoring are not fixed, but rather depend on how telemonitoring is organized and who is considered part of the collective for delivering it.

We suggest that addressing the different views on collective responsibility requires an ethical dialogue that supports the sensitivity and ownership of all individuals to their responsibility in the collective, and of the collective as such.^40,41^ A suitable approach for this, is responsive evaluation. It is a process-oriented method, which fosters dialogue among stakeholders across hierarchical levels and with differing views.^41^ We think that, before implementing and during use of telemonitoring, all actors of the collective, as well as health insurers, manufactures and policymakers should engage in such dialogues.^8,42^ The responsive evaluation should also clarify who comprises the collective, their shared goals, whom they are responsible to, and which normative standards guide their responsibilities.^43^ Further research is needed to assess how responsive evaluation could best support the appropriate implementation and use of telemonitoring and other digital health technologies that reshape collective roles and responsibilities.

### 5.2. Limitations

The following limitations of our study must be mentioned. First, since invitations were sent by email, participation required digital literacy, which is reflected in the high literacy scores. This suggests a selection bias that may have excluded patients with lower digital literacy. Future research should focus on including such patients, especially those disenrolled from telemonitoring due to low digital literacy. Second, this study aimed to explore the experienced roles and responsibilities of HCPs and patients using telemonitoring in general. These perceptions may of course differ between the care pathways (cardiac arrhythmia, resistant hypertension, and heart failure). However, given the limited number of assistants and nurses within the telemonitoring program (four and five, respectively), combined with the purposive sampling approach used in this study, stratification by care pathway was not feasible and fell beyond the scope of the present study. Consequently, our findings do not allow conclusions to be drawn regarding potential differences in viewpoints across specific care pathways. Future research could build on these findings by focusing on individual care pathways, which may provide further insight into pathway-specific differences and characteristics. Moreover, as we believe the ranking exercise was very feasible for respondents, future studies might consider a larger set of statements covering the topic in more detail, possibly allowing for more nuance in the results. Third, of the five respondents who were not significantly or uniquely associated with a single viewpoint, three were confounded, and two did not load on any viewpoint. The two respondents that did not load on any of the viewpoints could indicate that our study failed to identify additional viewpoints on experienced responsibilities in telemonitoring. However, inspection of the qualitative explanations of these respondents in their interviews does not show clear and coherent alternative views.

## 6. Conclusion

This study demonstrates that, in the context of cardiac telemonitoring, the views on roles and responsibilities of caregivers and patients differ, creating practical and ethical challenges. It is important to overcome these challenges in order to make optimal use of telemonitoring in clinical practice. While Q-methodology has previously addressed topics like digital health adoption and administrative responsibility, it had not been used to investigate moral responsibility in telemonitoring. We suggest that good use of telemonitoring requires an ongoing ethical prologue and dialogue, to sensitize stakeholders to different interpretations of responsibility especially among healthcare professionals. In particular, ethical dialogues should address the composition of collective responsibility, its goals, and the relationship between individuals and the collective. Furthermore, they should discuss the format and interpretation of patient-generated measurements, to ensure consistent and equitable care-delivery. Ethical dialogue sessions should be held prior to the implementation of telemonitoring, incorporated into caregiver training programs to foster ongoing reflection and improvement within existing telemonitoring programs, and lead to formalization of responsibilities in guidelines for caregivers.

## Supplemental material

Supplemental material - How telemonitoring impacts the responsibilities of patients and healthcare professionals in cardiology: A Q-methodology study Supplemental Material for How telemonitoring impacts the responsibilities of patients and healthcare professionals in cardiology: A Q-methodology study by Eva Meier, Marieke A. R. Bak, Job van Exel, Michiel M. Winter, Mariette van den Hoven, Tessel Rigter, Marlies P. Schijven in DIGITAL HEALTH

Supplemental material - How telemonitoring impacts the responsibilities of patients and healthcare professionals in cardiology: A Q-methodology study Supplemental material for How telemonitoring impacts the responsibilities of patients and healthcare professionals in cardiology: A Q-methodology study by Eva Meier, Marieke A. R. Bak, Job van Exel, Michiel M. Winter, Mariette van den Hoven, Tessel Rigter, Marlies P. Schijven in DIGITAL HEALTH

Supplemental material - How telemonitoring impacts the responsibilities of patients and healthcare professionals in cardiology: A Q-methodology study Supplemental material for How telemonitoring impacts the responsibilities of patients and healthcare professionals in cardiology: A Q-methodology study by Eva Meier, Marieke A. R. Bak, Job van Exel, Michiel M. Winter, Mariette van den Hoven, Tessel Rigter, Marlies P. Schijven in DIGITAL HEALTH

Supplemental material - How telemonitoring impacts the responsibilities of patients and healthcare professionals in cardiology: A Q-methodology study Supplemental material for How telemonitoring impacts the responsibilities of patients and healthcare professionals in cardiology: A Q-methodology study by Eva Meier, Marieke A. R. Bak, Job van Exel, Michiel M. Winter, Mariette van den Hoven, Tessel Rigter, Marlies P. Schijven in DIGITAL HEALTH

Supplemental material - How telemonitoring impacts the responsibilities of patients and healthcare professionals in cardiology: A Q-methodology study Supplemental material for How telemonitoring impacts the responsibilities of patients and healthcare professionals in cardiology: A Q-methodology study by Eva Meier, Marieke A. R. Bak, Job van Exel, Michiel M. Winter, Mariette van den Hoven, Tessel Rigter, Marlies P. Schijven in DIGITAL HEALTH

Supplemental material - How telemonitoring impacts the responsibilities of patients and healthcare professionals in cardiology: A Q-methodology study Supplemental material for How telemonitoring impacts the responsibilities of patients and healthcare professionals in cardiology: A Q-methodology study by Eva Meier, Marieke A. R. Bak, Job van Exel, Michiel M. Winter, Mariette van den Hoven, Tessel Rigter, Marlies P. Schijven in DIGITAL HEALTH

## Data Availability

The datasets generated and analyzed during the current study are available from the corresponding author on request.*
