# Supplementary material for: How telemonitoring impacts the responsibilities of patients and healthcare professionals in cardiology: A Q-methodology study
Source: Digit Health. 2026 Aug 28;12:20552076261484036. doi: 10.1177/20552076261484036 (PMC13527333; doi:10.1177/20552076261484036)
Supplement: Supplementary file 1 [file sj-pdf-1-dhj-10.1177_20552076261484036.pdf]

# Supplementary 1

## Table 1. Checklist for reporting a Q methodology study

| | Topic | Description | Check |
|---|---|---|---|
| **1.** | **Study design** | | |
| 1.1 | Source(s) of the concourse | To ensure a representative set of statements, we consulted a nurse and assistant from the telemonitoring program reviewed official caregiver protocols, and drew on findings from a scoping review on digital health and moral responsibility (Meier et al., 2024). This process revealed four themes: (1) shifting responsibilities to patients, (2) to nurses, (3) to physicians, and (4) the establishment of collective responsibilities among all actors, including developers. Based on these, the first author drafted 45 statements. | ✓ |
| 1.2 | Item sampling process | The initial set of 45 statements was refined with input from co-authors and a manager from HW and reduced to 25, and then piloted with a physician, nurse, and patient for comprehensiveness, clarity and feasibility. As a result of this structured sampling process, adjustments have been made to the wording and quantity of statements, resulting in a final set of 16 statements. | ✓ |
| 1.3 | Type of items | Written statements. | ✓ |
| 1.4 | Number of items | In this study we used 16 written statements. | ✓ |
| 1.5 | Pilot study | These statements have been piloted and tested for comprehensiveness, clarity and feasibility (i.e., duration of completing the ranking among a physician, nurse and patient). | ✓ |
| **2.** | **Data collection** | | |
| 2.1 | Selection of participants | Participants in this study were purposively sampled because our aim was to capture a diverse range of perspectives. | ✓ |
| 2.2 | Number of participants | A total of 30 individuals participated in our study, including patients, nurses, medical assistants, technicians, physicians and a manager of HW. | ✓ |
| 2.3 | Mode of interview | All participants took part in an individual interview. | ✓ |
| 2.4 | Type of interview | 20 interviews took place in person and 10 online. | ✓ |
| 2.5 | Ranking instruction | During the interview, participants read the statements aloud and ranked them on a forced-choice grid from 'strongly disagree' to 'strongly agree'. They then explained their reasoning for placing the statements. After completing the ranking, participants could review and adjust their choices. At the end, participants were asked to reflect on the statements they most strongly agreed or disagreed with and discussed how telemonitoring influenced their sense of role and responsibility. | ✓ |
| 2.6 | Sorting grid | The sorting grid consist of a 7-column forced-choice grid from 'strongly disagree' to 'strongly agree', with 1-2-3-4-3-2-1 places per column. | ✓ |
| **3.** | **Data analysis** | **Explanation** | |
| 3.1 | Factor analysis | The first step of the analysis is computing a correlation matrix showing the correlation of the ranking of the statements of each participant, indicating the strength of similarity between the 30 individual rankings of the statements. This is followed by factor analysis to identify clusters of respondents with similar rankings. We extracted the factors using principal component analysis (PCA). For the factor rotation we used varimax analysis to maximize high and low valued | ✓ |

| Topic | | Description | Check |
|---|---|---|---|
| | | factors (Zeller, 2005). Factor scores were calculated as weighted average rankings of statements per factor. Each factor array represents an idealized ranking pattern. | |
| 3.2 | Factor selection | To determine the number of factors, we combined Goldberg's (2006) method with qualitative interpretation (Appendix figure). When examining the correlations across 1- to 6-factor solutions, it becomes evident that factors in the 3-factor solution remained stable across the 4-, 5-, and 6-factor solutions (correlations > 0.80), while a distinct fourth factor emerged and remained consistent in the 5- and 6-factor solutions (correlation = .73). Qualitative data supported the coherence and relevance of this fourth factor. Based on this, we selected a 4-factor solution, which accounted for a total explained variance of 59%. | ✓ |
| 3.3 | Bipolar factors | Not applicable. | ✓ |
| 3.4 | Interpretation of the factors | The factors were interpreted based on their characterizing statements, which included the highest and lowest ranked statements, as well as their distinguishing statements, which were ranked statistically significantly differently from the other factors. To refine the interpretation, we incorporated interview data from participants significantly associated with each factor. | ✓ |
| 3.5 | Statistical software | KADE v1.3.0. | ✓ |
| **4.** | **Presentation findings** | | |
| 4.1 | Factor solution | Factor 1 accounts for the highest explained variance (21%). The remaining three factors exhibit lower explained variances of 15%, 14%, and 9%, respectively. 10 participants were statistically significantly and uniquely associated with the factors: 3 participants on factors 1 and 3 each, and 2 on factor 2 and 4. | ✓ |
| 4.2 | Factor arrays | See table 1 in manuscript. | ✓ |
| 4.3 | Distinguishing and consensus statements | See results section in manuscript. | ✓ |
| 4.4 | Description of the factors | See results section in manuscript. | ✓ |
