# Supplementary material for: How telemonitoring impacts the responsibilities of patients and healthcare professionals in cardiology: A Q-methodology study
Source: Digit Health. 2026 Aug 28;12:20552076261484036. doi: 10.1177/20552076261484036 (PMC13527333; doi:10.1177/20552076261484036)
Supplement: Supplementary file 2 [file sj-pdf-2-dhj-10.1177_20552076261484036.pdf]

Supplementary 2

Interview guide

5 min, Introductions

- Introduction about the research and explanation (stopping and pausing is always possible)
- Highlight the key points from the information letter (rationale, data processing)
- Duration (45–60 min)
- Give the voucher
- Ask if recording can start
- Turn on mobile and recording device

3 min, Consent form

- Have the participant read and sign the consent form (2 copies per person)?

3 min, Introductory questions

- Age?
- Highest level of education completed?
- What is their role within the telemonitoring program?
- How long have they been working there?

2 min, Explain the Q-grid and its format

Statements and questions

40 min, Statements

1) Every patient participating in telemonitoring program can understand the measurements taken.

- Is it important that the patient is able to do so? Why do you think so or not?
- If the patient needs to be able to understand the measurements, does responsibility for interpreting the measurements then shift from the healthcare provider to the patient?

2) The patient is responsible for taking good measurements (blood pressure, pulse, etc.).

- Why do you think so or not? Can the patient take good measurements without being able to interpret them?

3) The patient's loved ones are an important factor in the successful completion of measurements with the telemonitoring program.

- Why or why not? For which patients does this apply, and for which does it not?

4) Using telemonitoring makes the patient responsible for managing their own condition.

- How does the program make them responsible? What do you think about that?

5) The patient has the task of alerting the healthcare provider when a measurement is abnormal.

- Do you think it is good that they do or do not have this responsibility? Is it clear what is expected of the patient?

6) The patient must take a measurement at a fixed time. This gives them more responsibility.

- Does this make the patient more dependent on care? Less free? Why or why not?

7) The use of telemonitoring reduces the workload for nurses.

- How does the workload for the telemonitoring nurse change? Does telemonitoring have a positive or negative impact on this?

8) When telemonitoring is used, the nurse takes on more responsibility for medical decisions and the cardiologists takes on less.

- What tasks does the nurse currently have, and what tasks does the doctor have? Are these different from the workplace without telemonitoring? What do you think about that?

9) When measurements are abnormal, the nurse decides whether additional investigation is needed.

- How do you experience making this decision? Do you feel sufficiently supported to be able to make the decision?

10) The use of telemonitoring gives the nurse a different role in patient care.

- What does the nurse's role look like with telemonitoring? How does contact with patients change? What do you think about that?

11) It is the cardiologist's task to assess whether a patient can participate in telemonitoring.

- Are the factors used to assess whether a patient can participate clearly defined?

12) The use of telemonitoring reduces the cardiologists's workload.

- What causes this, or why not?

13) Through the use of telemonitoring is the cardiologist less involved with the patient.

- How does the patient–doctor relationship change? What do you think about that?

14) When the cardiologist can continuously see the patient's measurements, they have more responsibility regarding patient care.

- Measurements also come in during the weekend. Should someone be responsible for taking action on these?

15) An incorrect medical decision may be made. In that case, there is always one party (healthcare provider OR hospital OR developer) that is responsible.

- Is it always just one party that is responsible when something goes wrong?

16) If there is a fault in the telemonitoring measurement equipment and incorrect medical decisions are made as a result, healthcare providers are just as responsible as the developer.

- Can responsibility for a patient's care be shared between the doctor, nurse, and the patient themselves (and perhaps the developer)?

5 min, Wrap-up

- Ask whether the participant would like to receive the transcript and study results
- Check that you have their contact details
- Ask what the respondent thought of the interview
- Ask for any tips
- Thank them
- Take a photo of the sorted statements
- Turn off recording devices
