# Supplementary material for: How telemonitoring impacts the responsibilities of patients and healthcare professionals in cardiology: A Q-methodology study
Source: Digit Health. 2026 Aug 28;12:20552076261484036. doi: 10.1177/20552076261484036 (PMC13527333; doi:10.1177/20552076261484036)
Supplement: Supplementary file 3 [file sj-pdf-3-dhj-10.1177_20552076261484036.pdf]

Supplementary 3

Figure 1. Factor flow diagram

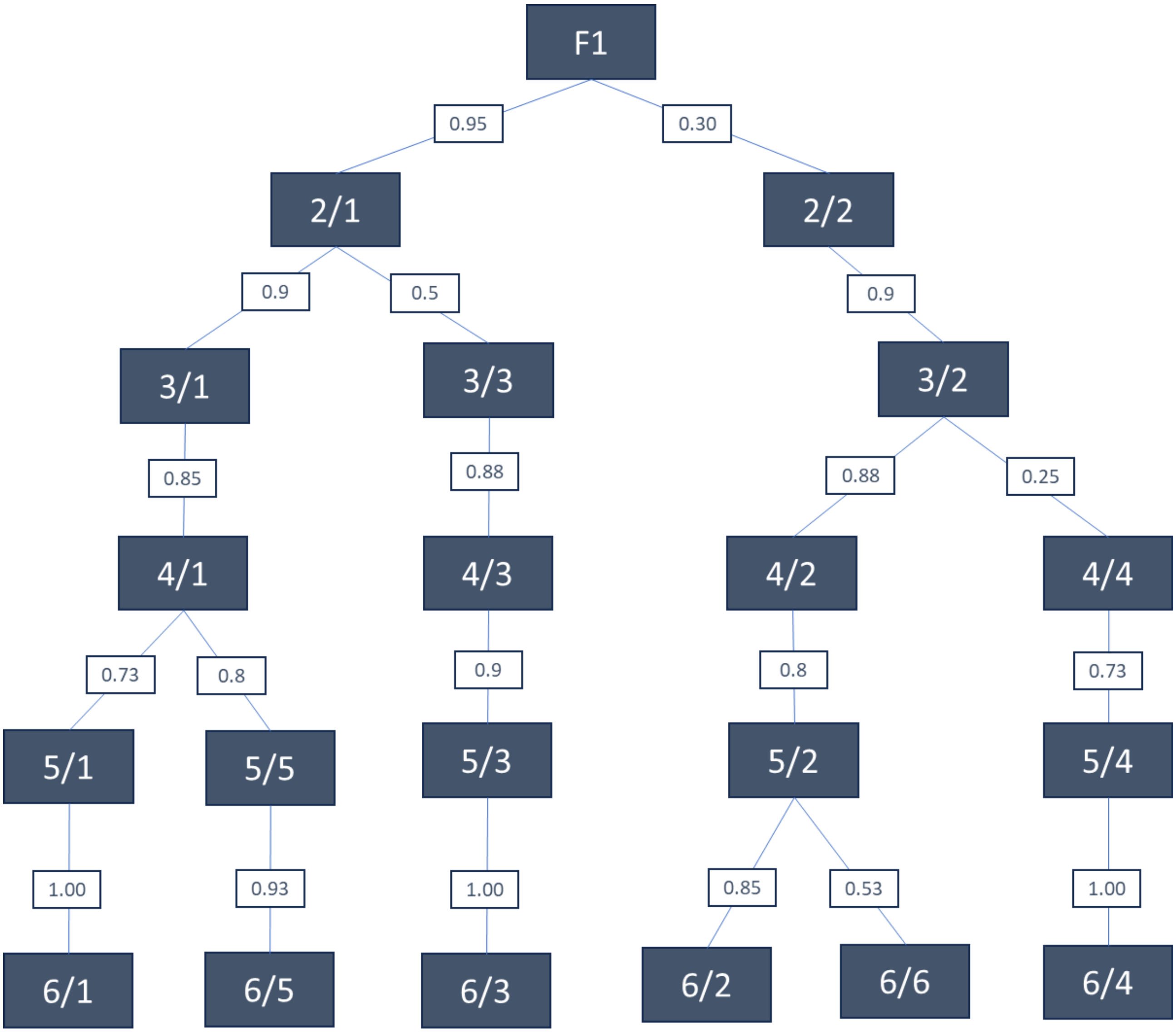

This factor flow diagram illustrates the correlations between Q-sorts for the 1- to 6-factor solutions. The rows represent the individual factor solutions, ranging from the 1-factor solution at the top to the 6-factor solution at the bottom. The connecting lines between the boxes highlight a selection of the highest correlations between different factor solutions, while the numbers within the boxes on the line indicate the specific correlation coefficients.
