# Supplementary material for: How telemonitoring impacts the responsibilities of patients and healthcare professionals in cardiology: A Q-methodology study
Source: Digit Health. 2026 Aug 28;12:20552076261484036. doi: 10.1177/20552076261484036 (PMC13527333; doi:10.1177/20552076261484036)
Supplement: Supplementary file 4 [file sj-pdf-4-dhj-10.1177_20552076261484036.pdf]

Supplementary 4

Table 2. Loading table defining variables for each factor indicated

Nm	Q-sort	F1	F2	F3	F4
3	Nurse1	0.8321	-0.2061	0.1178	0.1371
23	Car 7	0.7772	0.1576	-0.0681	-0.1470
9	As 4	0.7680	0.0547	0.2155	-0.0202
27	Car 9	0.6682	-0.1081	0.4656	0.1107
22	Car 6	0.6660	-0.2276	0.2505	0.0533
21	Pat 5	0.6558	-0.2561	0.2663	0.2442
14	Car 4	0.6468	-0.0136	0.0381	-0.1387
2	As 2	0.6242	-0.1076	0.0014	0.3039
6	Nurse 3	0.5838	0.1792	0.2806	0.4521
16	Pat 2	0.5702	0.1417	-0.1428	0.5572
26	Pat 6	0.5094	0.3409	-0.2892	0.2527
25	Tech 1	0.4965	0.0345	-0.2029	-0.0470
8	Manager	0.4331	0.1099	-0.0795	0.1437
15	Pat 1	-0.2346	0.7521	0.3017	0.1657
19	Tech 2	0.0568	-0.6843	-0.0143	0.2803
20	Car 5	0.4987	0.6512	-0.1329	0.0581
13	Car 3	0.1196	-0.2035	0.7693	-0.0276
24	Car 8	0.3459	0.2965	0.7591	-0.1526
28	Pat 7	-0.0301	0.0118	0.7340	-0.0994
7	Nurse 4	0.0572	0.0136	0.6987	0.0419
30	Pat 9	-0.1923	0.1430	0.6556	0.4279
18	Pat 4	-0.3269	0.0210	0.6287	0.4623
1	As1	0.3560	-0.4570	0.5797	-0.2824
4	Nurse2	0.1506	0.3523	0.5328	0.2550
10	Nurse 5	0.0664	0.1026	-0.1101	0.8659
11	Car 1	-0.0409	0.0828	0.1808	0.770
17	Pat 3	-0.0431	-0.3860	0.0741	0.7045
5	As3	0.4023	-0.2404	-0.1565	0.6997
12	Car 2	0.3791	0.0868	0.0617	0.5614
29	Pat 8	-0.0273	-0.0570	0.3798	0.4041
