# Supplementary material for: How telemonitoring impacts the responsibilities of patients and healthcare professionals in cardiology: A Q-methodology study
Source: Digit Health. 2026 Aug 28;12:20552076261484036. doi: 10.1177/20552076261484036 (PMC13527333; doi:10.1177/20552076261484036)

Supplementary 5

Figure 2. Factor Arrays

These figures visually illustrate the factor arrays of each viewpoint. It shows the average ratings of the 16 statements for each of the four viewpoints, compared to another viewpoint. Statement scorings positioned on the outer circle (+3) represent those most strongly agreed with, while scorings on the inner circle (−3) indicate those most strongly disagreed with.

Figure 2a. Visual representation of factor arrays of viewpoint 1: ‘Balancing Act’ and viewpoint 2: ‘Nurses: From Clinical Observers to Remote Monitors’

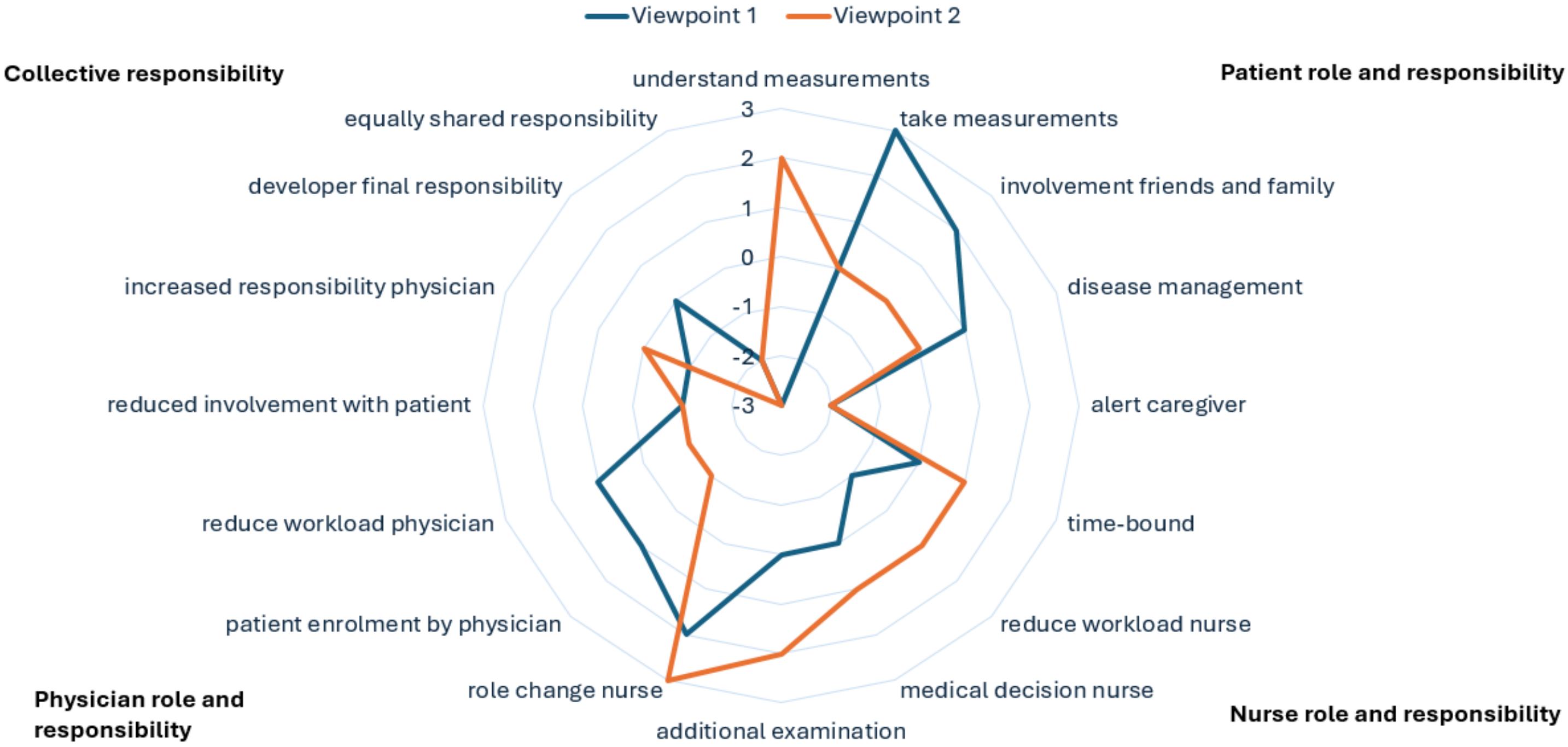

Figure 2b. Visual representation of factor arrays of viewpoint 1: 'Balancing Act' and viewpoint 3: 'Isolated Responsibility'

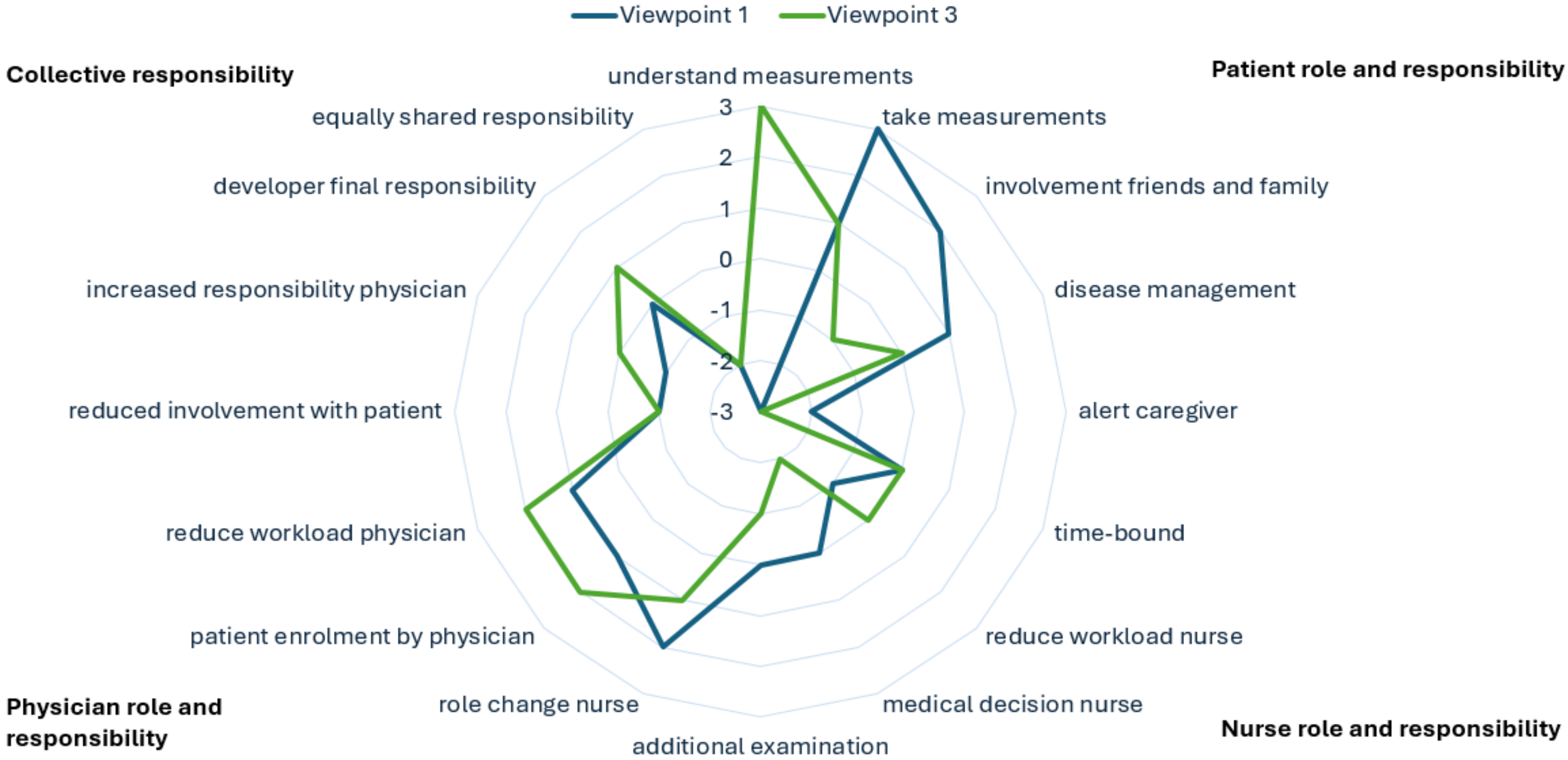

Figure 2c. Visual representation of factor arrays of viewpoint 1: 'Balancing Act' and viewpoint 4: 'Patients: Responsible but not Alone'

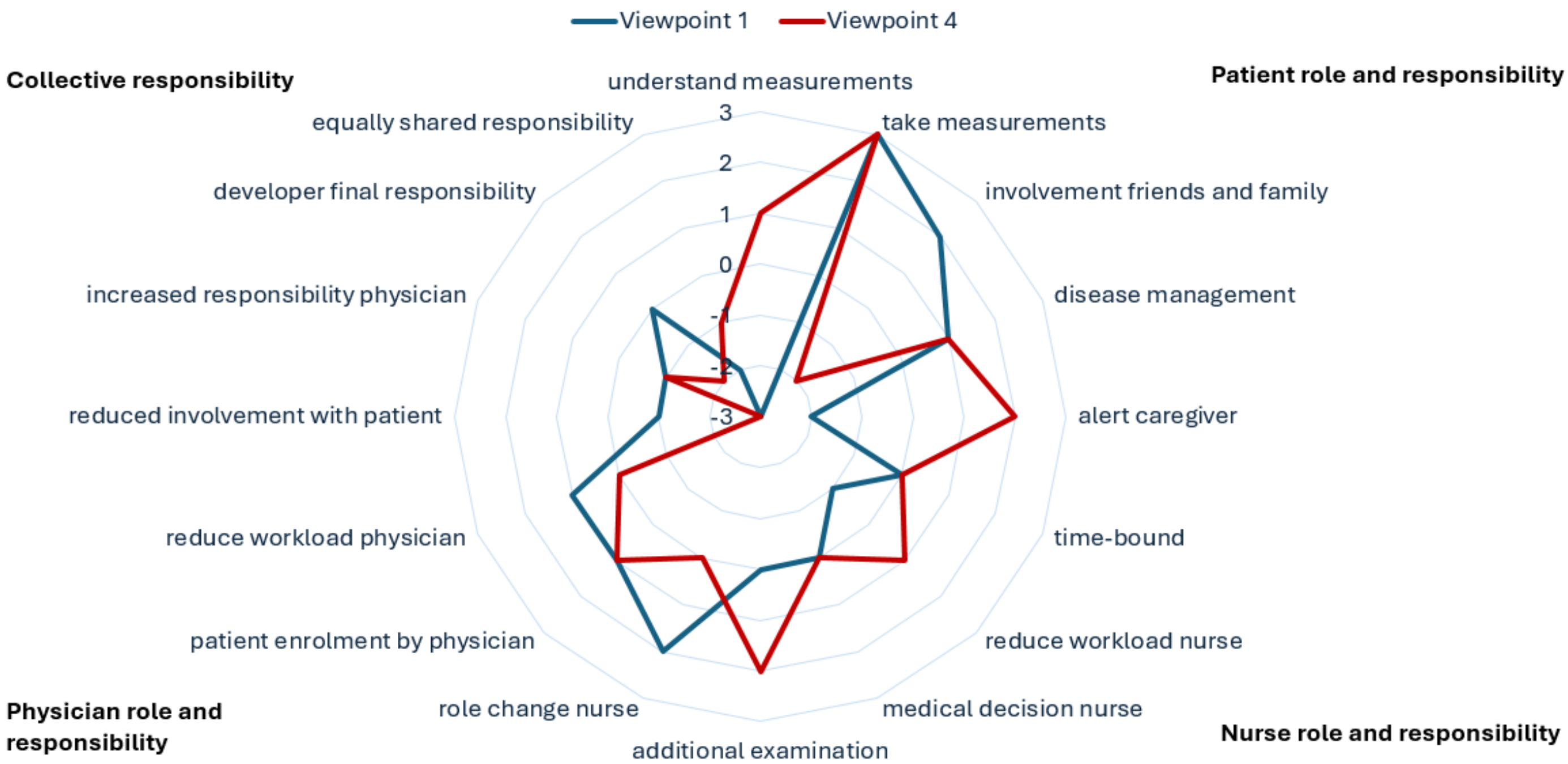

Figure 2d. Visual representation of factor arrays of viewpoint 2: 'Nurses: From Clinical Observers to Remote Monitors' and viewpoint 3: 'Isolated Responsibility'

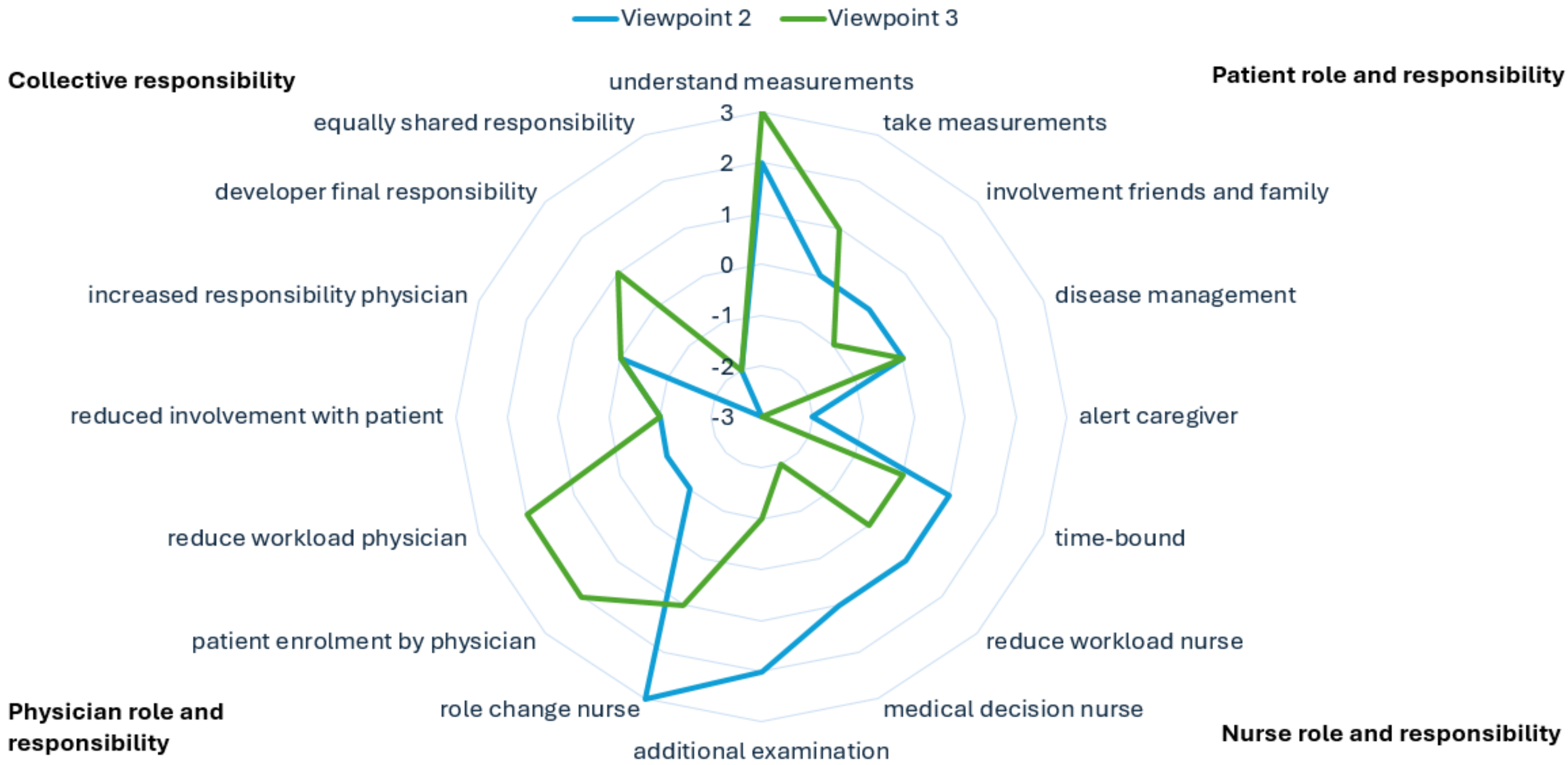

Figure 2e. Visual representation of factor arrays of viewpoint 2: 'Nurses: From Clinical Observers to Remote Monitors' and viewpoint 4: 'Patients: Responsible but not Alone'

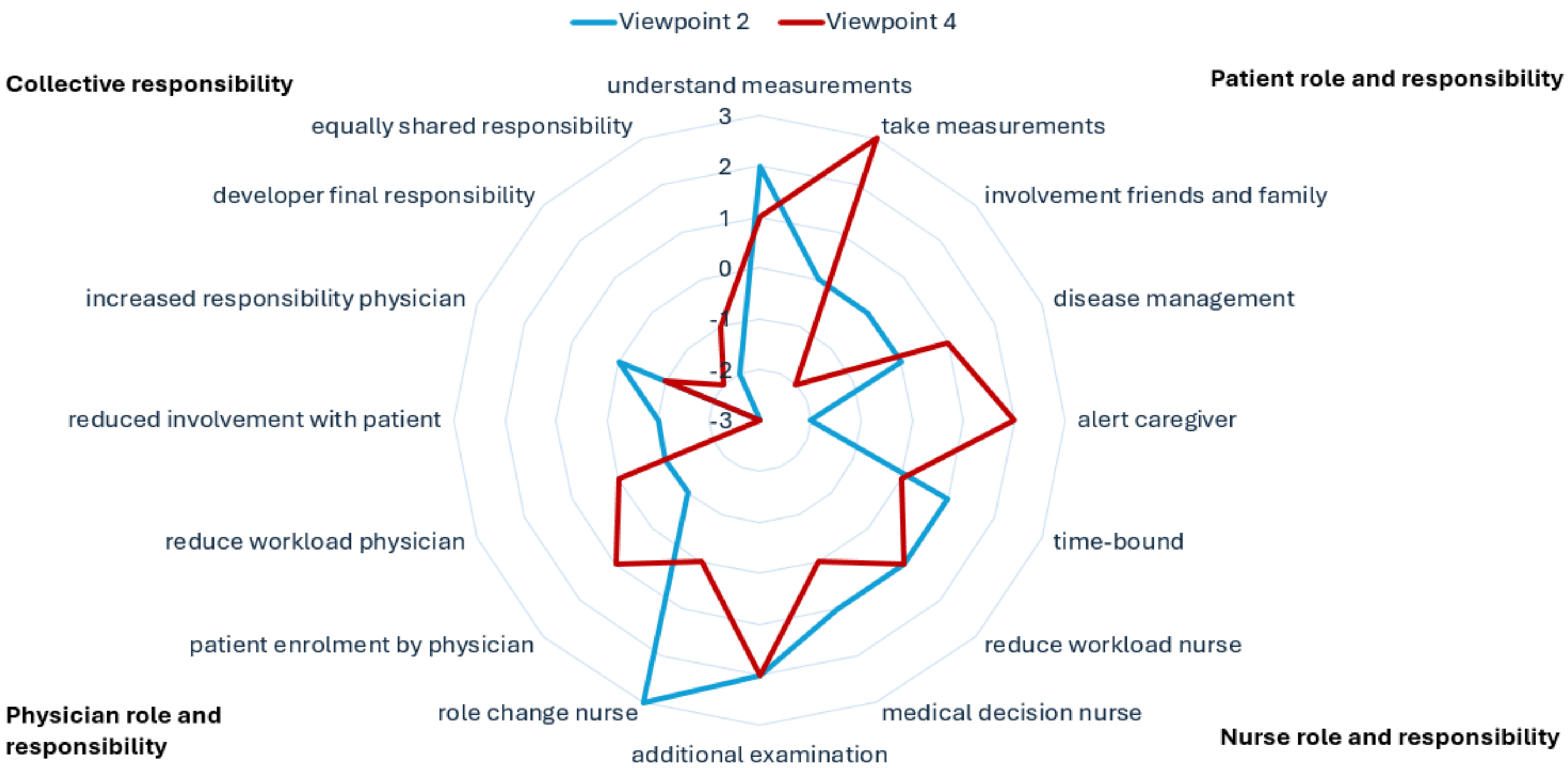

Figure 2f. Visual representation of factor arrays of viewpoint 3: 'Isolated Responsibility' and viewpoint 4: 'Patients: Responsible but not Alone'

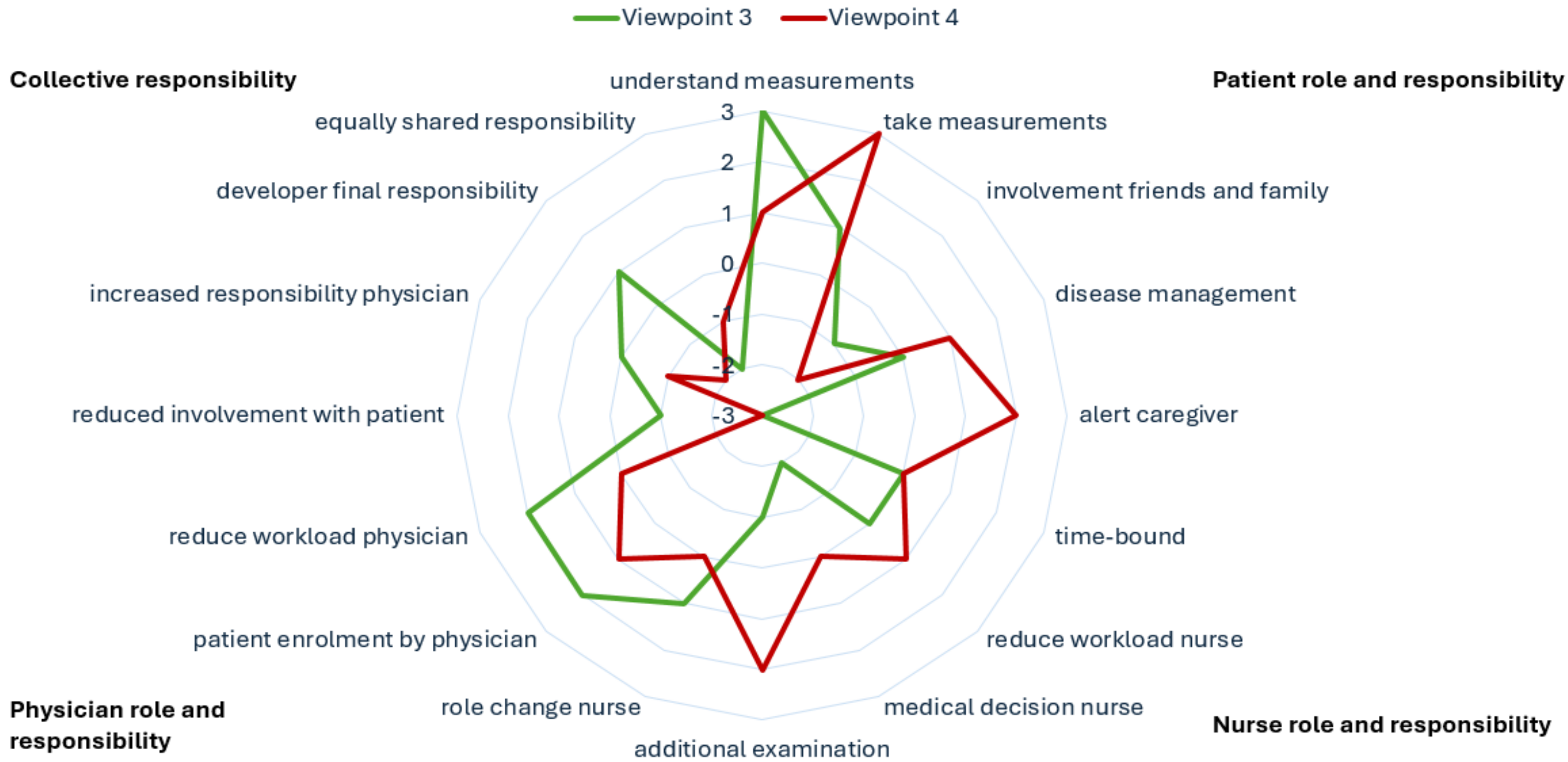

Supplement: Supplementary file 5 [file sj-pdf-5-dhj-10.1177_20552076261484036.pdf]
